# Bioinformatics and the Politics of Innovation in the Life Sciences: Science and the State in the United Kingdom, China, and India

**DOI:** 10.1177/0162243916631022

**Published:** 2016-02-24

**Authors:** Brian Salter, Yinhua Zhou, Saheli Datta, Charlotte Salter

**Affiliations:** 1 Department of Political Economy, King’s College London, London, UK; 2 Norwich Medical School, University of East Anglia, Norwich, UK

**Keywords:** politics, power, governance, markets/economies, other

## Abstract

The governments of China, India, and the United Kingdom are unanimous in their belief that bioinformatics should supply the link between basic life sciences research and its translation into health benefits for the population and the economy. Yet at the same time, as ambitious states vying for position in the future global bioeconomy they differ considerably in the strategies adopted in pursuit of this goal. At the heart of these differences lies the interaction between epistemic change within the scientific community itself and the apparatus of the state. Drawing on desk-based research and thirty-two interviews with scientists and policy makers in the three countries, this article analyzes the politics that shape this interaction. From this analysis emerges an understanding of the variable capacities of different kinds of states and political systems to work with science in harnessing the potential of new epistemic territories in global life sciences innovation.

## Introduction

The contribution of bioinformatics to state strategies on life sciences innovation has become an increasingly visible concern to governments. Announcing a £32 million investment in bioinformatics in February 2014, the UK Minister for Science David Willetts emphasized its “huge priority for government” and its “potential to drive research and development, increase productivity and innovation and ultimately transform lives” (Medical Research Council [MRC] 2014). His statement built on the promise of the *Strategy for UK Life Sciences* to make the United Kingdom “a world leader in genomics and bioinformatics” (UK Department for Business, Innovation and Skills [BISs] 2012, 41) and on the ambition stated by Jeremy Hunt, Secretary of State for Health, at the launch of Genomics England and the 100,000 Genome Project in July 2013 to make the United Kingdom “the first ever country to introduce this technology in its mainstream health system—leading the global race for better tests, better drugs and above all better, more personalized care to save lives” (Genomics England 2014). Meanwhile, in India, the Department of Biotechnology (DBT) is clear that the aim of its bioinformatics program and National Bioinformatics Network is “to ensure that India emerges a key international player in the field of bioinformatics; enabling a greater access to information wealth created during the post-genomic era and catalysing the country’s attainment of lead position in medical, agricultural, animal and environmental biotechnology” (India DBT 2014). This sense of national priority echoes the tone of DBT’s earlier strategy document *Bioinformatics policy in India*, which emphasizes that the requirements of innovation in science and technology mean that it is “of utmost importance that India participates in and contributes to the ensuing global bioinformatics revolution” (India DBT 2004, 3). And in China, the concern for keeping pace with global life sciences innovation through investment in bioinformatics is reflected in the projects funded in that field by the Natural Science Foundation of China (NSFC), the National High-tech Development Programme (863 Programme), the National Key Basic Research Development Programme (973 Programme), the National Science and Technology Major Projects, and the National Key R and D Technology Programme (Ai and Wang 2011; Wei and Lu 2008).

In terms of grand policy narratives, then, bioinformatics has come of age. States now see bioinformatics as a key component in life sciences innovation, in the pursuit of national advantage in the global knowledge markets of the future and in the servicing of the health needs of their populations. However, although they may agree on the importance of bioinformatics to the national interest, states disagree on how the value of its contribution to life sciences innovation can best be maximized. It is the purpose of this article to explore the politics of innovation that shape the differences in government strategies on bioinformatics. Central to this task is an understanding of the power relationship between science and the state, the different forms this relationship can take, and the impact of these differences on a state’s ability to support and exploit new epistemic domains such as bioinformatics.

The empirical vehicle for this analysis is the approach to bioinformatics adopted by the United Kingdom, China, and India. In the United Kingdom, we have an established player in the global competition for control of the future benefits of the life sciences, one accustomed to the nuances and difficulties inherent in the exploitation of its established science base. The situation of China and India is quite different. These are economies with an impressive track record in the penetration of existing global markets of established products but limited experience in the science-based anticipation of future markets through informed, but essentially speculative, state investment in emerging domains of the life sciences (Salter 2009a, 2009b). Unsurprisingly, this does not limit their ambition to challenge the Western hegemony in biomedical innovation, as their rapidly expanding commitment to the life sciences eloquently testifies. The question is how far their strategies on bioinformatics in support of this ambition are likely to influence the respective positions of the United Kingdom, China, and India in the global competition for advantage in the life sciences.

This article addresses three sets of questions. First, what is the contribution of bioinformatics to innovation in the life sciences, how has it developed, and what is its political value? What interests recognize this value and how have they sought to capture it by guiding the emergence of bioinformatics? Second, what is the contribution of the science–state relationship to the emergence of bioinformatics in the quite different political systems of the United Kingdom, China, and India? How and why does this contribution vary and what are the implications of this variation for a state’s ability to support and exploit new epistemic domains? Finally, given this analysis of the politics of bioinformatics, what is the balance of power between the three countries in terms of their ability to exploit the contribution of bioinformatics to life sciences innovation?

**Figure 1.**
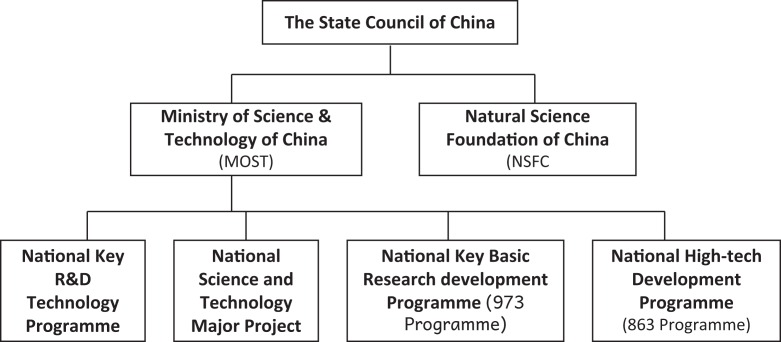
Bioinformatics policy and funding: China state structures. *Source:* The Ministry of Science and Technology of People’s Republic of China (http://www.most.gov.cn/eng/programmes1/index.htm) and National Natural Science Foundation of China (http://www.nsfc.gov.cn/).

**Figure 2. fig2-0162243916631022:**
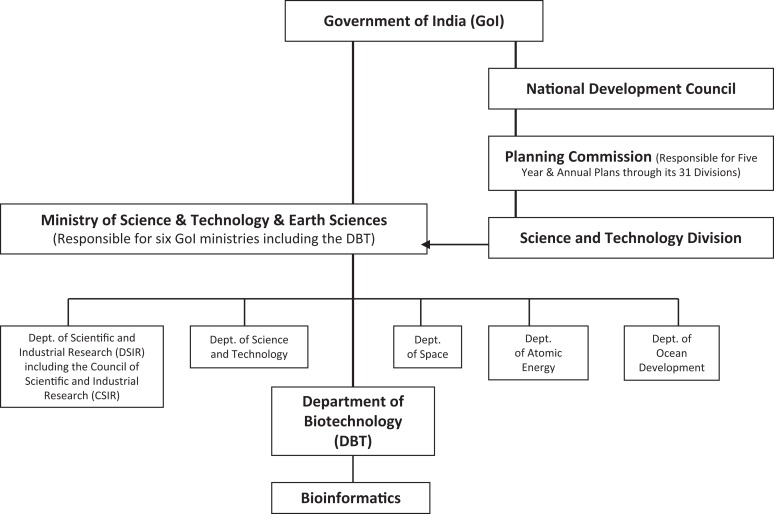
Bioinformatics policy and funding: India state structures. *Source:* Department of Science and Technology, Government of India (see http://www.dst.gov.in/>) and Planning Commission Government of India (see http://planningcommission.gov.in/aboutus/history/orgn.php?about=orgbody.htm).

To answer these questions, data were gathered in two phases. In the first, Internet desk-based scoping exercises of existing policies on bioinformatics in the three countries were conducted primarily through the analysis of policy documents of state organizations responsible for the field of science and technology (see Figures 1 and 2). In China, the focus was on publications of the State Council (e.g., National Five Year Plans, National Medium and Long Term Programme for the Development of Science and Technology, specific notices on bioindustry), the Ministry of Science and Technology (MOST—e.g., 973 and 863 Programmes), and the National Science Funding Council (NSFC—e.g., Five Year Plans); in India, on those of the Planning Commission (e.g., Five Year Plans), DBT (e.g., National Biotechnology Development Strategy reports), the Department of Scientific and Industrial Research, and the Department for Science and Technology (e.g., Working Group on Biotechnology annual plans); in the United Kingdom, on those of the Department of BISs (e.g., Office of Life Sciences reports), and the Research Councils (e.g., Biotechnology and Biological Sciences Research Council [BBSRC] and MRC annual reports). Supporting material was gathered from industry reports (e.g., Federation of Indian Chambers of Commerce and Industry [FICCI]), specialist reviews (e.g., Burrill Media), and statistical sources such as those of the National Science Foundation (NSF). The results of this phase were summarized in project working papers and used as the platform for the development of a semistructured interview schedule (Datta 2014; Zhou 2014). Thirty-two interviews were conducted with leading bioinformaticians, other elite scientists (particularly in the field of genomics), and policy makers from the state organizations listed above, evenly spread across the three countries. The distribution of the interviews by country and primary role (scientist or policy maker) is shown in Table 1(a). Often a scientist would have a secondary role and also act in a policy making capacity through formal membership of a state organization and, in some cases, would have strong industry links. The numerical effect of this overlap of roles within the interview sample is shown in Table 1(b). The interviews were recorded, transcribed, and analyzed employing the conceptual framework developed in the following two sections.

**Table 1(a).** Number of Interviews by Role and Country.

	China	India	United Kingdom	Total
Science	8	8	10	26
Policy	2	1	3	6
Total	10	9	13	32

**Table 1(b).** Number of Overlapping Roles by Country.

	China	India	United Kingdom	Total
Science	10	8	10	28
Policy	6	5	7	18
Industry	1	1	4	6
Total	17	14	21	52

## Biomedical Innovation, Bioinformatics, and Political Value

Generally conceived, innovation policies address the issue of how the state may best maximize the economic and social benefits of its scientific investment. Indeed, the rise to prominence of “innovation” as a policy domain owes much to its claim to be able to solve this thorny problem where others (e.g., “translation” policy) have failed. Hence, the European Union (EU) has its Innovation Union Initiative, United Kingdom its *Innovation Nation Policy*, the United States its *American Strategy for Innovation*, India its National Innovation Council, China its “new path of innovation with Chinese characteristics” [zi-zhu-chuang-xin], and so on (Salter 2013). In biomedical innovation, the policy issue is seen as one of how to facilitate the long, arduous, and uncertain process of scientific knowledge production from the basic science, through clinical experimentation and trials, to the therapeutic product. For example, the Cooksey report *A Review of UK Health Research Funding* concluded “that the UK is at risk of failing to reap the full economic, health and social benefits that the UK’s public investment in health research should generate” (the state interest) as a result of two key gaps. These are “translating ideas from basic and clinical research into the development of new products and approaches to treatment of disease and illness; and implementing those new products and approaches into clinical practice” (Cooksey 2006, 3).

It is in the context of public scientific investment as a risk endeavor that the political value of bioinformatics should be placed. Its political rise is a product of its perceived value to the process of biomedical innovation and the future markets to which such innovation gives access. As a discipline and epistemic domain, bioinformatics combines the knowledge, skills and techniques of biology, on the one hand, and computer science, statistics and mathematics, on the other (Lewis and Bartlett 2013; Luscombe, Greenbaum, and Gerstein 2001). In terms of its application, its territory is broad “covering anything from epidemiology, the modelling of cell dynamics, to its now more common focus, the analysis of sequence data of various kinds (genomic, transcriptomic, proteomic, metabolomic)” (Harvey and McMeekin 2002, 10). Behind its emergence lies the problem faced by biology when, from the 1980s onward, the volume, complexity, and variety of biodata production outstripped the discipline’s capacity to conceptualize, coordinate, analyze, and interpret it (Ouzounis and Valencia 2003). The fear of being overwhelmed was palpable and public (Butler 2001; Reichhardt 1999), with official bodies such as the US National Institute of Health Research recognizing that “the computers, algorithms, and software, let alone the support infrastructure, are not keeping up with the exponentially rising tide of data in biomedical research” (Botstein 1999). It is a concern that is still very much evident among our interviewees, who often said that “data generation kind of goes up quicker than computational power…essentially the bottle neck is not generating that data, it’s how to use that data” (Interview 4) and that “a new technology [such as sequencing or microarrays] comes out and then bioinformatics is just thrown in and has to somehow work out what to do with the new data that’s generated from it” (Interview 4). Part of the perceived problem is that it is structural, embedded in funding agency policy where “funding is not usually provided to help understand data, it’s provided to generate data” (Interview 2).

The problem has been particularly acute in the field of genomics where, fuelled by large government investment in projects such as the Human Genome Project (HGP) and skillful scientific public relations, expectations of this new field of “big science” (the HGP became known as the “Manhattan Project” of biology) were high but the promised benefits for public health remained distant (Galison and Hevly 1992; Lenoir and Hays 2000). With the biodata deluge generating more complexity and less clarity, something had to be done if genomic science was to maintain its impetus and access to public and private resources. Bioinformatics was presented as the epistemic and political answer. Hence, reports on genomics from the UK House of Lords Science and Technology Committee and Department of Health in the 2000s reiterate the difficulties faced by genomic medicine, the challenges to bioinformatics posed by the new genome technologies, the “painfully slow” translation of scientific research into “patient benefit,” the promise that, as Professor Dame Janet Thornton, Director of the European Bioinformatics Institute (EBI), put it: “it will be the biomedical informatics that will allow translations from knowledge and research into medical practice,” and the importance of investment in the research and training needs of bioinformatics (House of Lords Science and Technology Committee 2001, 2009; Department of Health 2003). In 2009, the Department of Health duly recognized that “The expansion in EMBL-EBI [European Molecular Biology Laboratory-European Bioinformatics Institute] data management capacity is vital in underpinning the sustainable development of the substantial investments in genetic, genomic and systems biology made by the Research Councils” (Department of Health 2009, 18). The formal political narrative was established with bioinformatics center stage.

As the public solution to a major problem in biomedical innovation, the position of bioinformatics in the policy narrative is secure. Yet at the same time, its epistemic identity in science remains fraught with political tensions. Integrating epistemic domains is a quintessentially political task because disciplines are constituted not only in terms of intellectual constructs and practices but also in terms of institutions with their particular interests and ambitions (Whitley 1976). Although the issue of how to deal with large amounts of biological data had been present since at least the 1980s, the impact of the importation of mathematical and computer science knowledge and skills into biology had initially been filtered through the existing power structures of biology; a process which rendered bioinformatics acceptable as a service function to the biological paradigm (Leonelli and Ankeny 2012, 29-31). Genomics changed all that because it is large, well-funded, highly complex and, most importantly, a state project that cannot be seen to fail. As a result, its political muscle is helping to reengineer the balance of power between the epistemic partners of bioinformatics. At the heart of this reconfiguration is the question of which epistemic paradigm should guide the organization and analysis of the biodata: mathematics or biology? In the initial stages of the partnership, it was assumed that mathematics and computer science would perform a data processing function guided by the hypotheses of biological theory. There appeared to be a natural convergence between the partners such that scholars described it as a “natural marriage,” albeit one where one partner was manifestly dominant over the other (Chow-White and Garcia-Sancho 2012, 14). More recently, this view of relationship development has been shown to be an overoptimistic interpretation of epistemic cohabitation. In its place has emerged a view of balance in the interdisciplinary production of bioinformatics and a recognition that it “will require some fundamental changes in biological assumptions on the part of biologists, and mathematical assumptions on the part of the “import” disciplines” (Harvey and McMeekin 2002, 21). In that happy situation, the new mathematical tools produced for analyzing biodata are then seen as both “the objects of knowledge production for the expert bioinformatician community and instruments for knowledge production for the wider molecular biology community” (Harvey and McMeekin 2007, 20). Bioinformatics performs a creative as well as a service function.

The political tensions inherent in this epistemic transition constitute part of wider shifts in the role of “big data,” as it has become known, in the scientific endeavor. The collection, storage, and analysis of very large data sets are not peculiar to biology. Indeed, compared to disciplines such as physics, chemistry, and climate science, biology is very much a late arrival in the big data domain (Mayer-Schonberger and Cukier 2013; Hey, Tansley, and Tolle 2009). Practices devoted to the extraction of inferences from data *in silico* have become sufficiently sophisticated that “computational tools for data analysis are assigned a prominent role in facilitating the extraction of patterns from data, while experimental work is conceived as means to verify and explain those patterns” (Leonelli 2012a, 50). The consequence is that the creative power in the interdisciplinary relationship moves to mathematics and computer science. The effect of this power transfer is to challenge the ways in which science is organized and practiced through the forms of collaboration, division of labor and integrative strategies (of models, data, theories, and software) set up to deal with the fact of big data. As a result, Leonelli claims, “Data-intensive methods are changing what counts as good science” (2012b, 2). As the bioinformatics space is progressively institutionalized, so new power roles are emerging to allow the benefits of the data bases to be exploited by a variety of global audiences. For example, curators act to create bioontologies, and adapt existing ones, in order to organize the data into a form capable of meeting the research needs of bioinformaticians and biologists alike (Leonelli 2012a, 58-59). In so doing, they are, as Farquhar and Sundar Rajan put it, engaging in “the rendering political of information, in and through its relationship with the database and archive” (Farquhar and Sunder Rajan 2014, 388).

Such is the significance of the power transfer that the traditional paradigm of hypothesis-driven research is being replaced by what has been termed “discovery science,” where the database is established first and the explanations of the patterns they contain follow later (Chow-White and Garcia-Sancho 2012, 146). Biology is becoming a “data-bound science” driven by the imperatives and logic of the database rather than by hypotheses derived from biological theory and applied to observation (Lenoir 1999, 35). In the workplace, the *in silico* “dry labs” of electronic databases and computation are becoming equally as important as the traditional *in vivo* “wet lab” as the primary location of disciplinary activity (BBSRC 2012). It is in this political space that the identity of bioinformatics is being forged. The evidence of our interviews suggests that the struggle continues within science for control of this political space and its strategic position in the territory of biomedical innovation. Tensions abound between the “laboratory style of reasoning” and the “statistical style of reasoning” employed in the work of wet and dry labs (Penders, Horstman, and Vos 2008, 749) and “the terms “data-driven” and “hypothesis-free” have become focal points of debates about the legitimacy of bioinformatics techniques and methods” (Stevens 2013, 66). There is no agreed definition of the bioinformatics identity but a strong awareness of the fact that the space exists, its scientific and political significance, the formative role of genomics and of the competing disciplinary ambitions for its future. How does the state then deal with both the potential and the uncertainty of this new territory?

## States, Science, and the Politics of Innovation

The competition between states for control of biomedical innovation is driven by the anticipated demand of future populations for improved and more efficient health care, the future knowledge market generated by this demand, and the economic benefits that will accrue to those able to shape access to that market to their advantage. In the bioeconomy as elsewhere, the advanced economies of North America and Europe met the uncertainties accompanying the shift from Fordist to post-Fordist modes of mass production and consumption with the evolution of the “competition” state as the vehicle for the pursuit of national advantage through innovation (Cerny 1997; Hay 2004). Rather than concerning themselves with government interventions to ensure full employment and respond to market failures, states began to focus their attention instead on the neoliberal supply-side policies that would give a sharp edge to their competitiveness in the global knowledge economy. Particularly in the case of the knowledge-driven bioindustries, this meant a concentration not only on the infrastructures of innovation but also on “agglomeration and network economies and the mobilization of social as well as economic sources of flexibility and entrepreneurialism” (Jessop 2002, 110). As a consequence, the competition states of the West have moved away from the national sponsorship of particular firms and technologies and toward policies designed to foster “the conditions necessary for innovation.” Rather than specific structural change, the competition state goal is seen to be one of stimulating a dynamic that enables the knowledge production process to become self-sustaining. As we shall see, the scientific community plays a key role in maintaining that dynamic.

While this analysis provides insights into the state’s likely role in life sciences innovation in the developed economies of the West, a different approach is necessary in the case of the emerging economies of the developing world. Focusing in the main on South Korea, Taiwan, Japan, and Singapore in the 1980s and early 1990s, the earlier work on the “developmental state” highlights its role in the promotion of rapid economic development through the targeting of particular industries with large global markets. The markets were already there. The political task was to penetrate them. To achieve this goal, the state protected its chosen industries using a range of policies such as import and credit controls, promoted them through state investment, guided private capital through incentive schemes, and measured their progress in terms of export achievements (Onis 1991). Backed by a strong, professional, and autonomous bureaucracy, the state sought to define the specific path of industrialization through the “government of the market” (Wade 2003). In this analysis, the essence of those states’ commonality is that they sought to challenge the control exercised by the developed world over the dynamic of globalization. If they were to access the wealth of global markets, if they were to “catch up” with Western countries, then the power of the state was required to make globalization work for them.

However, having caught up using the targeting of known markets as a primary policy objective, developmental states face the problem of “keeping up” in the context of future markets like those generated by the life sciences that are either unknown or decidedly uncertain (Weiss 2000). Like competition states, they are obliged to adapt their strategies of direct state intervention when faced with the innovation requirements of a science with a speculative future, an uncertain market, and a difficult path to commercialization (Lee and Schrank 2010). As a consequence, scholars have noted the evolution of developmental state governance into new forms described variously as the “adaptive state,” the “flexible state,” the “speculative state,” the “post-industrial developmental state,” the “transformative state,” and the “catalytic state” in their studies of Japan, China, India, South Korea, and Taiwan (Kim 1999; Salter 2009a; Wu 2004; Wong 2005). In seeking to move from borrowers to innovators in the life sciences, developmental states are obliged to review their *modus operandi* and the style of the bureaucracy that helps formulate and implement their innovation policies.

Central to the state’s role in life sciences innovation is a clear understanding of how the state relates to the scientific community and to the interests of that community. Like all enduring political arrangements, in the developed economies that relationship has historically been founded on an exchange of mutual benefits. Science supplies the state with a flow of knowledge that can enable the delivery of economic and social benefits to its citizens. The state supplies science with the resources to pursue its research interests. Supporting this core agreement is an infrastructure of embedded institutions and values designed to maintain the relationship’s authority and legitimacy; promote continuing engagement between the two partners; and facilitate the addition of new, mutually beneficial, and scientific dimensions to the agreement (Jasanoff 2004). Political exchange is continuous with scientists lending their expertise and authority to the activities of the state’s policy advisory system and the state facilitating and legitimizing science’s system of self-regulation (Jasanoff 1994). Although a permanent marriage, tensions undoubtedly exist within it and commentators differ in their interpretation of how these tensions alter its internal balance of power. In his work on the scientific elite of the United Kingdom and the United States, Mulkay emphasizes the power of the scientific elite, arguing that it “operates as a ‘buffer group’ [between science and state], successfully resisting instrumental demands from outside and maintaining considerable freedom for members of the academic research community to pursue their own ‘scientifically defined’ interests” (1976, 445). Here, the state sets the overall budget, but the scientific elite decides which area of science gets what. Others are skeptical of this view of scientific autonomy and present the state as the dominant partner who defines the scientific agenda in terms of the state’s political interest, and, in the case of the United States, uses science to legitimize government policies and programs (Mukerji 1989; Solovey 2001).

Interpretations of the balance of power between science and the state in developed economies may vary but all are agreed that the political relationship is one of mutual dependence where political resources such as finance, expertise, and decision-making are exchanged through an established complex of institutions, networks, and understandings. The situation in the emerging economies is quite different. On the one hand, the commitment to investment in science is clearly present. Between 2001 and 2011, the R and D investment of the economies of East, Southeast, and South Asia (including China, India, Japan, Malaysia, Singapore, South Korea, and Taiwan) increased far more rapidly than that of the West, with the result that their share of global R and D rose from 25 percent to 34 percent (NSF 2014, chapter 4). Much of this change has been driven by China, which has experienced a real annual growth in its R and D budget in this period of 18 percent, reaching US$208 billion in 2011, and making it the second highest in the world league table of R and D expenditure (NSF 2014, chapter 4). On the other hand, these impressive figures are not a product of joint science–state initiatives characterized by evenly balanced partnerships. Rather, at the outset, governments have certainly led and science has followed. The reasons are not hard to find. First, until recently, the developing countries did not see investment in science as a priority: they were concerned with existing not future markets. For example, China’s R and D investment in 1991 was 0.73 percent of gross domestic product rising to only 0.91 percent in 2001. The US equivalent was 2.72 percent for both years (NSF 2014, appendix table 4.13). Science, and most of all basic science, lacked political value—until the developmental state adopted innovation as its leitmotif in the late 1990s (Wong 2011). Second, and consequentially, the scientific community in such countries is still building its epistemic identity, institutions, status, and relationships with the state. In China, for example, a scientific elite is emerging, but it is inexperienced and lacks the characteristics normally associated with successful scientific communities such as self-regulation and promotion by merit (Cao and Suttmeier 2001; Suttmeier and Cao 1999). The implications of this relatively immature level of development in the scientific community are considerable. Science in the emerging economies lacks the political infrastructures for the internal and external management of epistemic change taken for granted in the West. While in the latter, the negotiations over who should benefit from the emergence of new epistemic territory such as bioinformatics may be tense, they are nonetheless handled within a set of institutions such as the UK research councils accustomed to (a) resolving allocation disputes internally and (b) translating the results into political demands on the state. Such mechanisms are largely absent in China and India with the result that the science–state relationship takes a quite different form.

Reinforcing these differences is a third factor: science is a transnational enterprise dominated by the West. The continuing migration of scientific labor from the developing to the developed countries reinforces existing scientific communities and constrains the formation of new ones (Hunter, Oswald, and Charlton 2009). At the India Institute of Science in 2005, 90 percent of those who finish PhDs chose to move overseas (Jayaraman 2005). In 2004, China’s Ministry of Personnel estimated that of about 580,000 students who had traveled abroad to study since the late 1970s, only 27 percent had returned (Li et al. 2004). Lacking the political muscle derived from the historic relationship with the state enjoyed by Western scientific communities, the scientific elites of the emerging economies remain largely supporting players in the politics of global science, with their entry to the transnational scientific networks contingent upon their attractiveness as potential partners in collaborative research (Wagner and Laydesdorff 2005). Thus, in a sense, it can be said that developmental and competition states have done what they have always done. The former have used bureaucracy and targeted finance to build innovation capacity in the future markets of science, the latter have relied on their historic dominance of the global knowledge markets through the transnational power of their scientific elites to persuade key elements of that capacity into the scientific jurisdictions of competition states. How far is this true of bioinformatics?

## State Strategies in China, India, and the United Kingdom

A simple structural comparison of the state organizations with the responsibility for supporting the development of bioinformatics in China, India, and the United Kingdom reveals some initial and instructive differences (Figures 1 [Fig fig2-0162243916631022]–3). In China and, to a lesser extent, India, departments of state play the dominant role in the formulation and execution of policy on bioinformatics. In the United Kingdom, on the other hand, although the Department of BIS controls the overall size of the budget, the details of bioinformatics policy are worked out at the level of the research councils where the scientific community is the dominant influence. The top-down style of innovation governance of the developmental state is most obvious in China, where the State Council sets the agenda across policy domains through its five-year plans for economic development. The relevant subordinate departments, in the case of science and technology policy, the MOST and the NSFC, then faithfully interpret that agenda within their established funding programs and show where and how they will deliver the policy goals laid down by the State Council. MOST deals mainly with large applied and product-oriented projects of US$4 to US$5 million and the NSFC with basic research of less than US$100,000. In India, likewise, the five-year plans of the Planning Commission, though less rigidly enforced than in China, provide the priority setting framework for the MOST and the DBT with the latter holding the primary responsibility for bioinformatics.

**Figure 3.**
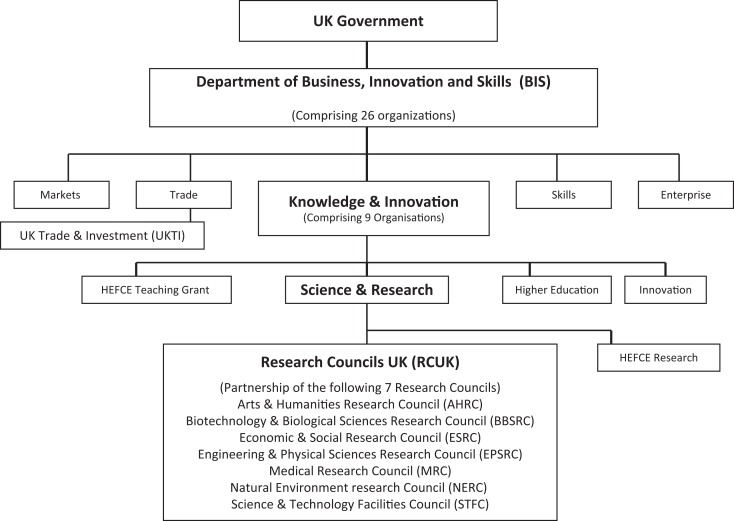
Bioinformatics policy and funding: UK state structures. *Source:* Guide to Business, Innovation and Skill 2012-2013 (https://www.gov.uk/government/uploads/system/uploads/attachment_data/file/34764/12-p120c-guide-to-bis-2012-2013.pdf).

Using these plans as a policy tracking tool, we can see that the significance of bioinformatics was first recognized in India with the launch of the Biotechnology Information System network by DBT in 1986 “to create an infrastructure that enables it [India] to harness biotechnology through the application of bioinformatics” (DBT 2014). A decade later in China, bioinformatics first makes its appearance in the 9th Five Year Plan of MOST’s National High-tech Development Programme (863 Programme) with the commitment in 1996 to fund a project on the “Development and Establishment of a Database for Bioinformatics” and a center for bioinformatics within the College of Life Sciences at Beijing University, with the intention that it should act as the official mirror site for major international biological databases (Wei and Yu 2008, 1). Thereafter, an analysis of the five-year plans of the relevant state agencies of both China and India show the continuing presence of lists of projects apparently designed to enhance the bioinformatics capacity of the two countries through the creation of databases, clusters, networks, and skills (Datta 2014; Zhou 2014). In the decade up to 2014, the total funds committed were £303 million in China and £19 million in India (Tables 2 and 3).

**Table 2(a).** China: MOST Funding of Bioinformatics (2005-2014).

Year	Scheme	Category	Funding (£ million)
2005	The National Program for Sci-Tech Basic Conditions Platform Construction during the Year of 2004 to 2010		0.3
2006	863 Programme	Bioinformation and computational biological technology	8.0
2007	863 Programme	Bioinformation and computational biological technology	6.5
2008	863 Programme	Bioinformation and computational biological technology	2.0
	863 Programme	Biological and medical technology-genome-wide association study and pharmacogenomics study on common severe diseases	20.0
	Eleventh five-year National Key Technology R&D Plan	Key technology development and demonstration of public information share and exchange for biotechnology industry	3.0
	Second call for eleventh five-year plan National Science and Technology Major Project	New drug creation and development (2009-2010)	216.0
2010	2011 National Science and Technology Major Project	New drug creation and development	10.0
2011	2012 National Science and Technology Major Project	New drug creation and development	N/A
2013	863 Programme (2014)		20.0
2014	863 Programme (2015)	Biological and medical technology—key technology of biological big data development and application	N/A
Total			285.8

*Source:* The Ministry of Science and Technology (MOST) of the People’s Republic of China (http://www.most.gov.cn/).

*Note:* N/A = Not applicable.

**Table 2(b).** China: NSFC Funding of Bioinformatics (2005-2013).

Year	Funding (£ million)
2005	0.9
2006	1.0
2007	0.9
2008	0.8
2009	1.2
2010	0.7
2011	2.4
2012	3.3
2013	3.6
Total	14.8

*Source:* National Natural Science Foundation of China (NSFC; isisn.nsfc.gov.cn).

**Table 3.** India: Department of Biotechnology Funding of Bioinformatics (2005-2014).

Year	Funding (£ million)
2005-2006	1.7
2006-2007	2.3
2007-2008	2.1
2008-2009	2.1
2009-2010	1.2
2010-2011	2.2
2011-2012	1.9
2012-2013	2.0
2013-2014	2.5
Total	18.0

*Source:* Datta (2014, annex 1).

To understand the significance of these figures, it is necessary to place them in their structural context. In the case of the apparently large Chinese investment, guiding the allocations are the policies of the State Council geared entirely to economic needs not to the needs of a fledgling epistemic domain. For example, bioinformatics is included as an industry servicing agent in the Council’s *Some policies promoting the development of bio-industry* (2009), *Decision of the State Council on Accelerating the Fostering and Development of Strategic Emerging Industries* (2010), and *Notice of the State Council on Issuing the Bio-Industry Development Plan* (2012; State Council of the PRC 2009, 2010, 2012). Acting within this frame, MOST takes the same approach to bioinformatics, for example, in its *12th Five Year Plan for the Medical and Pharmaceutical Industry* (MOST 2012a). Such an approach leads to call specifications directly linked to bioindustry such as the £3 million call of the 973 Programme in 2008-2010 for three projects: “The Development of Database for Bio-technology and Industrial Information,” “The Standardization, Integration and Application of Bio-technology and Industrial Information,” and “The Grid-based Key Technology and Software for Bioinformation” (MOST 2012b, 43).

As a result, as shown in Table 2, over 70 percent of bioinformatics funding is for specific application-oriented research provided by the industry-oriented MOST. Within this, the majority funding (£216 million of the total £285.8 million) is via the applied “New Drug Creation and Development” scheme (Nature 2010). In contrast, China’s agency for the funding of basic research—the NSFC—plays a minor role in state support for bioinformatics characterized by the funding of a plethora of small projects (£14.8 million 2005-2013). Reflecting on the nature of bioinformatics funding, a senior manager of the Beijing Genomics Institute (BGI) commented that apart from the three calls for the “Bioinformation and Computational Biological Technology” scheme of MOST’s 863 Programme, “there are basically no funds for this discipline [bioinformatics]…but some relevant bioinformatics projects can win support every year” (Interview 9).

Meanwhile, in India, the Five Year Plans of the Planning Council have, since their inception in 1951, focused on how the economic interest of the country can best be served by the policies of the departments of state. By the early 2000s, the narrative of the Plans in the field of science and technology had become the pursuit of “global leadership.” Hence in the *Tenth Five Year Plan 2002-2007*, for example, bioinformatics was selected as one of the fields that was “expected to be all pervasive and have far-reaching impact” in India’s bid to become a global leader, building on the nation’s experience in IT and pharmaceuticals (Planning Commission 2002, 10.159). Similarly, in *Biotechnology: A Vision*—*Ten Year Perspective*, the guiding document for the DBT for the 2001-2010 period, genomics and bioinformatics are listed as the first two biotech areas for development (DBT 2001). What is less clear from the voluminous policy documentation of the Planning Commission and the DBT is how this objective is to be achieved in terms of its necessary engagement with the scientific community. Mention is routinely made of the need for infrastructure elements such as workforce training, new bioinformatics courses, the establishment of bioinformatics institutes, and international linkage, but how these are to be integrated in the absence of a guiding scientific paradigm of epistemic change remains opaque (see, e.g., DBT 2007, 2014; Working Group on Biotechnology 2011). Furthermore, there are radical shifts in state enthusiasm for the bioinformatics project. Thus, the extensive discussion of bioinformatics in the *Eleventh Five Year Plan 2007-2012* is oddly matched by its very limited presence in the *Twelfth Five Year Plan 2012-2017*, where there are proposals on the expansion of computerized databases of patient records but little mention of the development of the field as a whole. Even the routine report in the *Twelfth Plan* on the *Eleventh Plan’s* achievements fails to mention “bioinformatics” (Planning Commission 2013, BOX 8.4: 250)

As with China, construction of a new bioinformatics epistemic identity is clearly not the state’s objective since this would require an explicit scientific conceptualization of how state support for particular epistemic qualities of bioinformatics can enable the translation of genomic knowledge into health-care products. Rather, there is an assumption that the components of such support are self-evident and only need to be listed in order to have the desired effect (Datta 2014; Zhou 2014).

The evidence from our China and India interviews strongly suggests that this deficiency is the result of, on the one hand, the state’s failure to engage and recruit relevant sections of the scientific community and, on the other, the inability of science itself to formulate a coherent epistemic view of how bioinformatics should be incorporated into biomedical innovation. The approach adopted to bioinformatics development in these states appears to be an outcome not of a scientific understanding of the needs of biomedical innovation (which itself is an outcome of epistemic political bargaining within science) but of the state’s interpretation of what those needs might be, given its preoccupation with economic development as the driving organizational principle. Disaffection with this approach is most evident in China, where interviewees point to the failure to establish a national center for bioinformatics as a symbolic example of the state’s insensitivity to demands from the scientific community. One leading bioinformatician described how scientists from the Chinese Academy of Sciences originally petitioned MOST for a national bioinformatics center in 1999, but to little effect. The suggested explanation provides a flavor of the state–science relationship.

Chinese officials don’t know the importance of a national center for scientific research in China. They think that a new national center is a kind of waste because international databases are open access to Chinese scientists. Another reason is about leadership. More and more Chinese universities and institutes are conducting bioinformatics research and establishing their own bioinformatics center. Which university or institute, or who, can be the leader of this large project? China won’t take any action until we find a proper answer to this question. (Interview 25)

As the interviews make clear, the problem for Chinese scientists is that without a national bioinformatics center they lack the political muscle to integrate their many and various domestic bioinformatics activities, then to engage on equal terms with the major international databases of the West and Japan in terms of setting the agenda and direction for their development. The United States’s National Center for Biotechnology Information (NCBI), the United Kingdom’s EBI, and the DNA Databank of Japan (DDBJ) constitute core elements in the global infrastructure of bioinformatics and a “dominant, hegemonic presence” to which China has only conditional access (Harvey and McKeekin 2010, 492). China does have the BGI with a global operation and offices in the United States, United Kingdom, Japan, and Denmark. However, as the interview with a senior BGI manager made clear, unlike the NCBI, EBI, and DDBJ, its business model is based solely on the supply of bioinformatic services to science and industry, not on the active furtherance of bioinformatics as a discipline through the provision of a platform for international research, the organization and promotion of data sharing, and professional training (Interview 15). For example, BGI has a the “Green Super Rice” project funded by the Bill and Melinda Gates Foundation that provides a sequencing service to other grantees of the Gates Foundation and is a partner in the Genome 10K Consortium of Scientists (G10KCOS—China Daily 2013; G10KCOS 2015). China’s leading bioinformatics center BGI engages readily with the international scientific community and global bioinformatics markets, but it does so on a reactive rather than proactive basis, lacking the capacity to act as a promotional national center for China’s own bioinformatics development.

Indian scientists are equally concerned about the absence of a national bioinformatics center and the fact that there is “no common platform where all the data can reside together and people can join to do analysis and collaborate with people for analysis” (Interview 26). India does have specialist institutes such as the India Statistical Institute engaging in bioinformatics research but, as with BGI, not the promotion of the discipline itself. Part of the problem are differences between state organizations regarding the appropriate model to be used in fostering biomedical innovation. As one clearly frustrated interviewee put it:

The Ministry of Health has a different approach [to biomedical innovation]. Within the Ministry of Science and Technology, CSIR, which is a department in itself, has a different approach. DBT has a different approach, and DSD has a different approach. And then you have the Ministry of Commerce which has a different approach. (Interview 27)

One noticeable effect of this fragmentation of direction at the state level is the lack of fit between bioinformatics skills training in India and the advanced needs of genomics-based biomedical innovation such as dealing with very large data sets (Interview 14). Similar views were expressed by Chinese scientists, often placing their comments in the context of the absence of a national bioinformatics center that could and should act as a focus for research-linked skills training comparable to that provided by the United Kingdom’s EBI.

The presence of a national bioinformatics center in the United Kingdom since 1994 and not, thus far, in China and India reflects the balance of power in the science–state relationship in the three countries. That balance of power is in itself a product of the ability, or otherwise, of the scientific community in the three countries to deal with internal epistemic change and, if successful, then drive forward the resulting agreement. The United Kingdom’s European Molecular Biology Laboratory–EBI is Europe’s hub for big data in biology (EBI 2014). It exists because “science has brought these things together,” scientists “have had to organize themselves in terms of how they co-ordinate together,” and European research “works through a bottom-up approach” (Interview 22). Contrast this with the situation in China and India where the identity of the new discipline remains one where the biosciences, lacking the historic capacity to set the epistemic agenda with the state, simply allow computing science to contribute a service rather than a creative function to the interdisciplinary relationship. A leading Chinese bioinformatician commented: “Many people recognize the significance of bioinformatics for studying bioscience as an instrumental discipline, but fail to see or value its existence and development as a discipline itself” (Interview 10). In the United Kingdom, with science driving the process of change in bioinformatics through the internal politics of its scientific community, an underlying scientific paradigm has been developed to guide and legitimize that change—one that is absent in the state dominated initiatives of China and India. Hence, we find that the BBSRC’s annual reports over the last decade not only place a growing emphasis on bioinformatics but also conceptualize this change in particular ways. For example, the 2012 report *Bioscience for Society. A Ten Year Vision*, having noted with approval, the exponential growth of experimental data and the increasing use of *in silico*–based modes of research, develops a concept of “predictive biology” with experimental data, models, and bioinformatics tools at its center (BBSRC 2012, Figure 1).

The epistemic construction of the new disciplinary identity has been matched by a continuing search by the UK scientific community for resources from a variety of public and private resources. Thus, the EBI is located on the Wellcome Trust Genome Campus in Cambridge and is funded by the Wellcome Trust, the BBSRC, MRC, EU, European Member States, National Institutes of Health (NIH), the European Molecular Biology Organization, and the pharmaceutical industry. As this list implies, running the EBI is an internationally competitive business with other national bioinformatics centers the main rivals. In this context, the support of the UK state for EBI bids for international resources such as those of the EU is a significant advantage (Interview 22). At the same time, with scientific interests defining the agenda, the institutional expression of those interests across the research councils has been politically aligned through a division of funding labor between the BBSRC, MRC, Engineering and Physical Sciences Research Council (EPSRC), and NERC and their distinctive contributions to the development of bioinformatics made explicit through a Cross-Council Funding Agreement (see EPSRC 2014). The result is a steadily increasing level of research council funding for bioinformatics totaling £163.9 million since 2005 (Table 4).

**Table 4(a).** United Kingdom: BBSRC Funding of Bioinformatics (2005-2014).

Year	Category/theme	Funding (£ million)
2005	Bioinformatics	0.0
2006	Bioinformatics and biological resources fund pilot	6.4
2008	Bioinformatics and biological resources	5.5
2009	Bioinformatics and biological resources	6.7
2010	Bioinformatics and biological resources	7.1
2011	Bioinformatics and biological resources	5.5
2012	2011-2013 Tools and resources development fund call 2	1.9
2012	Bioinformatics and biological resources	6.6
2012	Tools and resources development fund call 2 (bioinformatics tools and computational approaches to the biosciences)	1.5
2013	Bioinformatics and biological resources	6.0
2014	Bioinformatics and biological resources	6.5
Total		53.7

*Source:* Data from “*BBSRC 20 Years of Pioneering*”: http://www.bbsrc.ac.uk/web/FILES/Publications/anniversary-brochure.pdf. Biotechnology and Biological Sciences Research Council (BBSRC): http://www.bbsrc.ac.uk.

**Table 4(b).** United Kingdom: MRC Funding of Bioinformatics (2012-2015).

Year	Category/theme	Funding (£ million)
2012	MRC/Biotechnology and Biological Sciences Research Council systems immunology of the human life course	3.0
2012	Initiatives in informatics research	19.0
2013	Initiatives in informatics research	20.0
2014	Initiative in medical bioinformatics	39.1
2015	Initiative in medical bioinformatics	10.9
Total		92.0

*Source:* Medical Research Council (MRC): http://www.mrc.ac.uk.

**Table 4(c).** United Kingdom: EPSRC Funding of Bioinformatics (2013).

Year	Category/theme	Funding (£ million)
To present	Biological informatics	14.2

*Source:* Engineering and Physical Sciences Research Council (EPSRC): http://www.epsrc.ac.uk/research/ourportfolio/researchareas/bioinformatics/.

**Table 4(d).** United Kingdom: NERC Funding of Bioinformatics (2012-2019).

Year	Category/theme	Funding (£ million)
2012-2019	Mathematics & informatics for environmental omic data synthesis	4.0

*Source:* Natural Environment Research Council (NERC): http://www.nerc.ac.uk.

Given that UK science has both an agenda and a plan for the development of bioinformatics, the role of the UK state in pursuit of national advantage becomes one of facilitating that agenda through financial and political support at national and international levels. With regard to the latter, it has a head start over its Chinese and Indian competitors because of the global hegemony of Western states in the life sciences. Originally propelled by the HGP and HapMap projects, the creation of global institutions supporting databases by Western states rendered “genomics a selectively global industry, creating a specific map determined by Western science, technology, and government and economic interest” (Thacker 2006, 18). Control of the databases ensures that Western science set the rules both for their operation and for the requirements of access to them. Hence, there is a much lower chance of incorporation of data from less prestigious, non-English-speaking laboratories in developing countries and less chance of the scientists from such countries participating in the development of international databases (Leonelli 2014, 10). One leading Chinese bioinformatician described how he was still waiting for access after applying to the NCBI database of Genotypes and Phenotypes four years ago (Interview 23).

What Harvey and McKeekin have termed the “political economy of self-regulation in bioinformatics” serves to fuel the continuing evolution of fresh forms of governance regarding quality, standards, and norms by the international scientific community. They cite the proliferating range of bioinformatics tools developing standards for harmonizing the “ontologies” of data in diverse databases through organizations such as the Microarray Gene Expression Data Society, the Macromolecular Structure Database as part of the worldwide Protein DataBank, and the Gene Ontology Consortium project (Harvey and McKeekin 2010, 502). Such examples of the institutional controls continuously generated by Western science illustrate the hegemonic dynamic of bioinformatics governance which began with the creation of the Bermuda rules in 1996. Attended by the Wellcome Trust, the NIH National Center for Genome Research, the US Department of Energy, the HGP of Japan, the German HGP, the UK MRC, and the European Commission, this meeting set out the new rules for the deposition of genomic data as a precondition for international collaboration between contributing laboratories to the HGP (Harvey and McKeeking 2007, 55). Since then, Western transnational networks of science have constructed through their communities of experts a political architecture of bioinformatics self-regulation with which Chinese and Indian scientist are obliged to collaborate on Western terms. If they do not accept the standards embedded in this hegemony, they will not get published (Interview 14).

Chinese and Indian scientists recognize the fact of Western dominance in bioinformatics and typically see their development in this field as behind the global pace, describing themselves as “4-5 years behind the West” (Interview 18—China) and “we’re always laggards” (Interview 14—India). A director of a Chinese genomics research center commented: “Bioinformatics in China is still at a relatively early stage, with few internationally influential articles, databases, algorithms, and software. The collaboration between bioinformatics research and experimental biology is not adequate” (Interview 11). From the UK perspective, although bioinformaticians interviewed would frequently have collaborations with scientists in the United States, Europe, and Japan via common databases and networks, their collaboration with China and India is, at best, described in terms of potential and the provision of advice rather than regular interaction with equal partners. From this imbalance between developed and developing countries then stems the frustration of Chinese and Indian scientists with what they see as their governments’ failure to fight their corner, documented earlier. A further difficulty for China and India is that the hegemony rests not just on the global reach of the Western scientific community but also on the market infrastructure that supports it. Bioinformatics in the developed world engages with a vibrant industry anxious to provide both services and creative input to the translation of genomic data into clinical utility (Harvey and McKeekin 2007). EBI has an organizational arm devoted to the cultivation and maintenance of such relationships, including an EBI-bioinformatics industry “club” that meets four times a year to exchange views and develop agendas (Interview 22). And the newly established Genomics England, although a government initiative, is contracting out much of its bioinformatics work to private industry in its project to bring biological and clinical data together (Genomics England 2014). By contrast, India has a small bioinformatics sector constituting barely 2 percent of the biotech sector (FICCI 2012), and geared mainly to low level, routine bioinformatics services and not to the needs of advanced research (Interview 25). Similarly, China’s bioinformatics industry is, as a director of a Chinese university bioinformatics department put it, “small scale and low level,” focusing on the processing of bioinformatics data with little capacity for “challenging research work” (Interview 10).

## Conclusions

As a case study of an emergent knowledge territory, bioinformatics provides important insights into the internal dynamic of science, the form of its relationship with the state, the variations in that relationship across political systems, and its contribution to the national and transnational politics of innovation in the life sciences. No one doubts that science has power through the exercise of epistemic control. What this article shows is that the exercise of that power is contingent upon its ability to identify, shape, and deliver on the needs of the state on a continuing basis. In the case of the life sciences, the governments of China, India, and the United Kingdom are unanimous in their belief that bioinformatics should supply the link between basic research and its translation into health benefits for the population and the economy. Yet at the same time, as ambitious states vying for position in the future global bioeconomy, they differ considerably in the strategy adopted in pursuit of this goal. As the nature of the science–state concordat varies, so does the ability of a state to exploit the opportunities offered by emerging epistemic territories.

At the political heart of these differences lies the interaction between epistemic change within the scientific community itself and the objectives and apparatus of the state. In the United Kingdom, although there are continuing tensions in bioinformatics between the epistemic domains of mathematics and computer science, on the one hand, and biology, on the other, they are tensions which have been institutionalized and managed through the scientific community’s control of the research councils and access to private funding bodies such as the Wellcome Trust. Led by genomics and driven by the political imperatives it has generated, science has recruited the United Kingdom’s competition state to a strategy that neatly blends scientific interest, national ambition, and population benefit into a convincing vision of the future. The state, for its part, is able to delegate to science the thorny political issue of how to maintain the United Kingdom’s position in the global competition for advantage in life sciences innovation. With the state acting as facilitator and providing appropriate political and financial support, science then takes responsibility for the delivery of a common agenda. The customary concordat between science and state is thus maintained.

In contrast, in China and India, the science–state engagement takes a quite different form with different results. Both states lack an established and self-confident scientific community with the capacity to define its own agenda for the development of bioinformatics, relate that agenda to the needs of the state, and advance it through the institutions of a mutual concordat. Rather, India’s concordat is premised on personal rather than institutional networks and China’s is too one-sided in favor of the state to be described as a balanced political contract. Given the nature of the science–state relationship in the two countries, there is no obvious mechanism to facilitate negotiations between the epistemic partners of mathematics and biology in order to produce a new discipline of bioinformatics capable of energizing life sciences innovation. Individual scientists have taken the initiative in India, but these have not cohered into a plausible strategy. In China, scientists are unaccustomed to defining the future scientific agenda and so await guidance from a state apparatus that lacks the expertise to construct it. In both countries, the absence of clear leadership from science has left the developmental state to launch a series of policy initiatives in bioinformatics backed by no clear conceptualization of their combined contribution to life sciences innovation. By default, China and India have adopted a model where bioinformatics continues to perform a service function to biomedical science rather than a creative function to biomedical innovation.

Confronted by the hegemony of a Western science sustained in the field of bioinformatics through a powerful global network of databases, scientific organizations, governance, and supporting markets, both science and state in China and India are obliged to wait in the wings for the opportunity to participate in the bioinformatics revolution as supporting actors. Lacking the ingredients of a science–state concordat to challenge this hegemony, they are obliged to recognize the reality of a global politics of life sciences innovation where power is embedded through the historic control of epistemic territory. Their experience is almost certainly not confined to the life sciences. Given the variable capacity of scientific communities to construct and present their agenda for new epistemic domains to the state, coupled with the historic differences between countries in the institutional efficiency of the science–state relationship, it can be anticipated that other fields of science will be equally subject to the nuances of this political dynamic.
